# Activating transcription factor 4 mediates adaptation of human glioblastoma cells to hypoxia and temozolomide

**DOI:** 10.1038/s41598-021-93663-1

**Published:** 2021-07-08

**Authors:** Nadja I. Lorenz, Alina C. M. Sittig, Hans Urban, Anna-Luisa Luger, Anna L. Engel, Christian Münch, Joachim P. Steinbach, Michael W. Ronellenfitsch

**Affiliations:** 1 Dr. Senckenberg Institute of Neurooncology, University Hospital Frankfurt, Goethe University, Schleusenweg 2-16, 60528 Frankfurt am Main, Germany; 2 German Cancer Consortium (DKTK), Partner Site Frankfurt/Mainz, Frankfurt am Main, Germany; 3 Frankfurt Cancer Institute (FCI), University Hospital Frankfurt, Goethe University, Frankfurt am Main, Germany; 4 University Cancer Center Frankfurt (UCT), University Hospital Frankfurt, Goethe University, Frankfurt am Main, Germany; 5 Institute of Biochemistry II, Goethe University, Frankfurt am Main, Germany; 6 Cardio-Pulmonary Institute, Frankfurt am Main, Germany

**Keywords:** Cancer, Cell biology

## Abstract

The integrated stress response (ISR) is a central cellular adaptive program that is activated by diverse stressors including ER stress, hypoxia and nutrient deprivation to orchestrate responses via activating transcription factor 4 (ATF4). We hypothesized that ATF4 is essential for the adaptation of human glioblastoma (GB) cells to the conditions of the tumor microenvironment and is contributing to therapy resistance against chemotherapy. ATF4 induction in GB cells was modulated pharmacologically and genetically and investigated in the context of temozolomide treatment as well as glucose and oxygen deprivation. The relevance of the ISR was analyzed by cell death and metabolic measurements under conditions to approximate aspects of the GB microenvironment. ATF4 protein levels were induced by temozolomide treatment. In line, *ATF4* gene suppressed GB cells (ATF4sh) displayed increased cell death and decreased survival after temozolomide treatment. Similar results were observed after treatment with the ISR inhibitor ISRIB. ATF4sh and ISRIB treated GB cells were sensitized to hypoxia-induced cell death. Our experimental study provides evidence for an important role of ATF4 for the adaptation of human GB cells to conditions of the tumor microenvironment characterized by low oxygen and nutrient availability and for the development of temozolomide resistance. Inhibiting the ISR in GB cells could therefore be a promising therapeutic approach.

## Introduction

With a median survival of less than one year in unselected cohorts, glioblastoma (GB) represents the most frequent, malignant primary brain tumor in adults^1^. So far, standard therapy is palliative and consists of surgical resection followed by alkylating chemotherapy with temozolomide and radiotherapy and optional alternating electrical fields for patients stable after radiochemotherapy^2,3^. For the improvement of existing therapy schemes, new approaches are urgently needed.

One reason for the poor prognosis of GB is therapy resistance—either tumor cells do not even initially respond to therapy (primary resistance) or develop resistance during the course of treatment (secondary resistance). In addition to therapeutic interventions, tumor cells are also challenged by a local decline of oxygen and nutrient supply in the GB microenvironment. Thus, the selective pressure favors tumor cells capable of mounting an effective stress response to enable adaptation and ensure cell survival. The integrated stress response (ISR) is a conserved adaptive program that can be activated by distinct stress signals including ER stress caused by the accumulation of unfolded proteins in the endoplasmic reticulum (unfolded protein response, UPR), hypoxia as well as glucose and amino acid deprivation^4–7^. While the ISR is primarily a pro-survival cell response, in some cellular models chronic or severe stress has also been reported to induce cell death^5^. Activation of the ISR is accomplished by one of four eIF2α kinases (PERK, GCN2, PKR and HRI) with distinct regulatory domains and activating triggers^7,8^. Activation of these kinases results in phosphorylation of the eukaryotic translation initiation factor 2α (eIF2α) which in turn leads to a reduction in global translation initiation and facilitates preferential translation of selected mRNAs, including the core effector of the ISR activating transcription factor 4 (ATF4)^5^. Thus, ATF4 is frequently used as a marker for ISR activation^5,9^. ATF4 induction has been demonstrated in consequence of hypoxic conditions, ER stress as well as glucose and amino acid deprivation^6,8–12^. In GB ATF4 has not yet been studied in the context of hypoxia and combined glucose deprivation which characterize the conditions of the tumor microenvironment.

Via its leucine zipper region, ATF4 interacts with other transcription factors and activates an array of target genes including genes involved in amino acid synthesis, angiogenesis, metastasis, differentiation and drug resistance^9^. The termination of the stress response is regulated by a negative feedback loop that leads to dephosphorylation of eIF2α mainly by GADD34^9^.

ATF4 expression has been reported to be upregulated in different types of human cancers and overexpression correlated with earlier tumor progression and therapy resistance^13^. Pharmacological inhibition of the ISR could be an opportunity to sensitize tumor cells to conditions of the tumor microenvironment and increase their sensitivity towards existing therapy strategies. Previous studies demonstrated that PERK inhibition itself can reduce tumor cell survival but is also associated with severe side effects in vivo especially on pancreatic function^14^. The small molecule inhibitor ISRIB blocks the ISR downstream of P-eIF2α by targeting eIF2B, which is required as guanidine nucleotide exchange factor in the translation initiation process and thereby sustains global protein translation^15–17^.

Few studies on ATF4 in GB have been conducted and indicate an elevated expression in comparison to normal brain tissue^18^. Also in the context of chemotherapy, ATF4 has been suggested to interfere with autophagy^19,20^.

While induction of ATF4 by typical conditions of the GB microenvironment with severe hypoxia and low nutrient supply^21,22^ is plausible, the relevance of ATF4 in this context has not been investigated in detail yet^23^. Similarly, effects on clonal survival, the main readout to evaluate temozolomide effects in vitro have not been performed^18,20,24^.

In this experimental study we investigated the regulation of ATF4, the main orchestrator of the ISR, under conditions of the glioma microenvironment. Hypoxia and temozolomide chemotherapy induced ATF4 mRNA expression and protein levels. Using pharmacological and genetic approaches we found that ATF4 directs tumor cell protective responses to mediate therapy resistance. This included protection from combined hypoxia-/nutrient restriction-induced cell death as well as from temozolomide chemotherapy. Therefore, ATF4 could be a promising candidate for therapeutic inhibition.

## Results

### ATF4 is induced by pharmacological ER stress activators, glutamine depletion and temozolomide treatment in human GB cell lines

**Figure 1:**
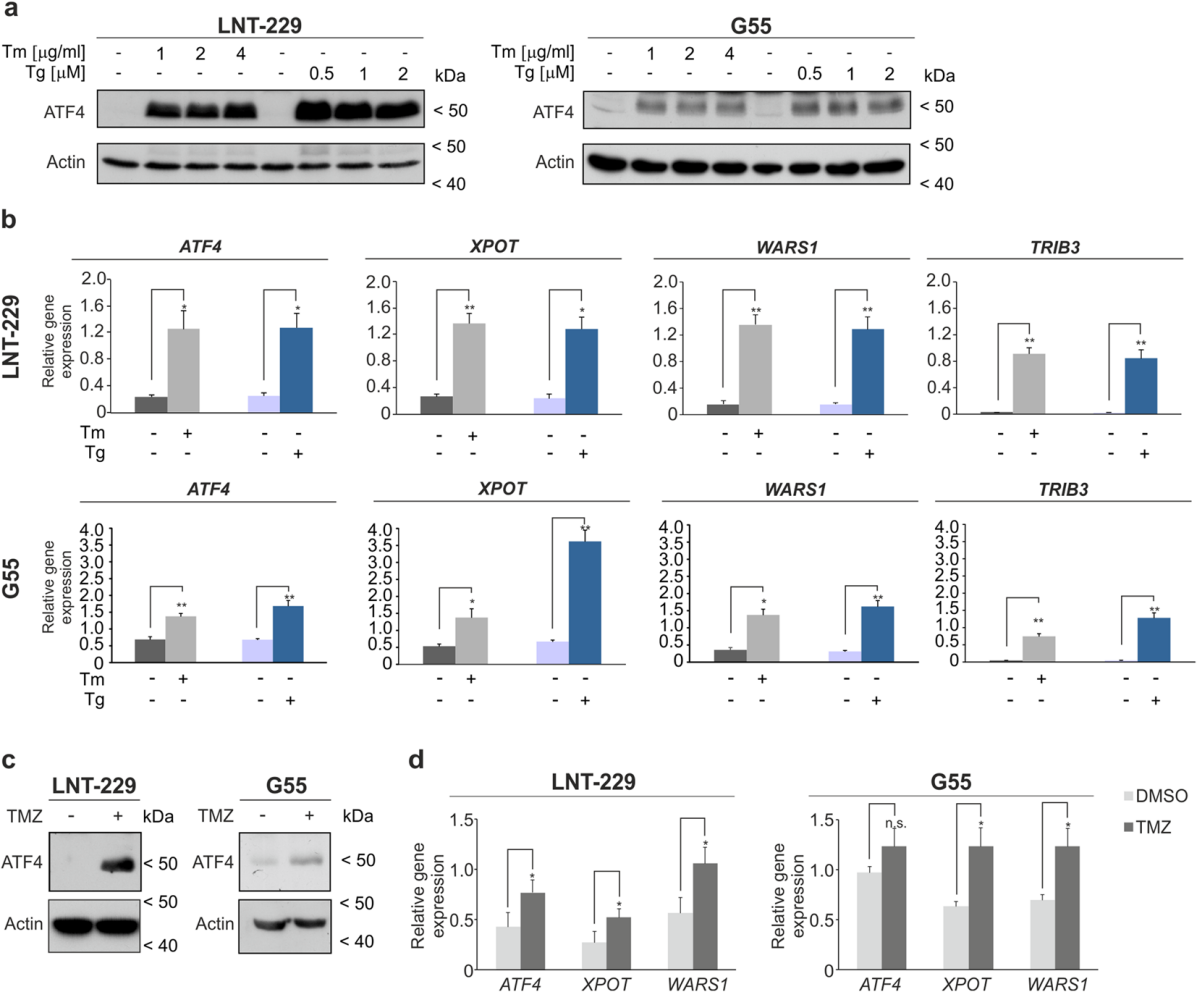
ATF4 is regulated by pharmacological ER stress activators and is induced by TMZ treatment in human glioblastoma cells. (**a**) LNT-229 and G55 cells were incubated with increasing concentration of thapsigargin (Tg) and tunicamycin (Tm) in serum-free DMEM for 8 h. Cellular lysates were analyzed by immunoblot with antibodies for ATF4 and actin. (**b**) LNT-229 and G55 cells were treated with 2 µg/ml tunicamycin (Tm) or 1 µM thapsigargin (Tg) for 8 h in serum-free DMEM. cDNA was analyzed for the expression of *ATF4* and its target genes *XPOT*, *WARS1* and *TRIB3* by qPCR. *18S* and *SDHA* were used as housekeeping genes for normalization (n = 3, mean ± SD, *p < 0.05, **p < 0.01, Student’s *t* test). (**c**) LNT-229 and G55 cells were treated with 400 μM temozolomide (TMZ) in serum-free DMEM for 24 h. Cellular lysates were analyzed by immunoblot with antibodies for ATF4 and actin. (**d**) cDNA was analyzed for induction of *ATF4* and target genes *XPOT* and *WARS1* by qPCR (n = 3, mean ± SD, n.s. not significant, *p < 0.05, Student’s *t* test). *SDHA* and *18S* were used as housekeeping genes for normalization.

To investigate the importance of the ISR in human GB cell lines basal *ATF4* mRNA expression levels were analyzed (Fig. S1a). Based on these results one GB cell line with low and one with high *ATF4* mRNA expression, LNT-229 and G55 respectively, were chosen for further experiments. *ATF4* mRNA levels of primary GB cells (NCH690, NCH644 and NCH421K) were comparable to the expression levels of GB cell lines. For comparison with non-tumor cells human astrocytes were investigated. Here, under basal conditions *ATF4* mRNA levels were also similar to the expression levels of primary GB cells (Fig. S1a). ER stress was pharmacologically induced in LNT-229 and G55 cells with tunicamycin or thapsigargin. Treatment led to reduced cell growth after 24 h and even more pronounced after 96 h incubation (Fig. S1b). Pharmacological activation resulted in an upregulated ATF4 expression on protein level in LNT-229 and G55 cells in all tested concentrations (Fig. 1a). Additionally, both ER stress inducers led to an increased mRNA level of *ATF4* itself as well as its target genes *XPOT*, *WARS1* and *TRIB3*^8,25–28^ (Fig. 1b).

ATF4 is known to be a major regulator of the ISR under amino acid deprivation conditions^10^. Glutamine withdrawal alone was sufficient to induce ATF4 expression in LNT-229 and G55 cells; concomitant glucose restriction did not result in an additional induction of the transcription factor (Fig. S1c, S1d). Serum withdrawal in the presence of glutamine had no effect on ATF4 expression (Fig. S1c). *ATF4* itself as well as the ATF4 target genes *XPOT*, *WARS1* and *TRIB3* were transcriptionally upregulated during glutamine starvation in LNT-229 and G55 cells (Fig. S1e).

The anti-glioma chemotherapeutic temozolomide has been reported to increase ATF4 expression as a consequence of UPR induction^18,20^. In both, LNT-229 and G55 cells treatment with temozolomide resulted in an increased ATF4 expression compared to vehicle treated cells and a transcriptional upregulation of *ATF4* and its target genes *XPOT* and *WARS1* (Fig. 1c,d).

### Gene suppression of *ATF4* inhibits activation of the integrated stress response under pharmacological ER stress induction and glutamine deprivation

**Figure 2:**
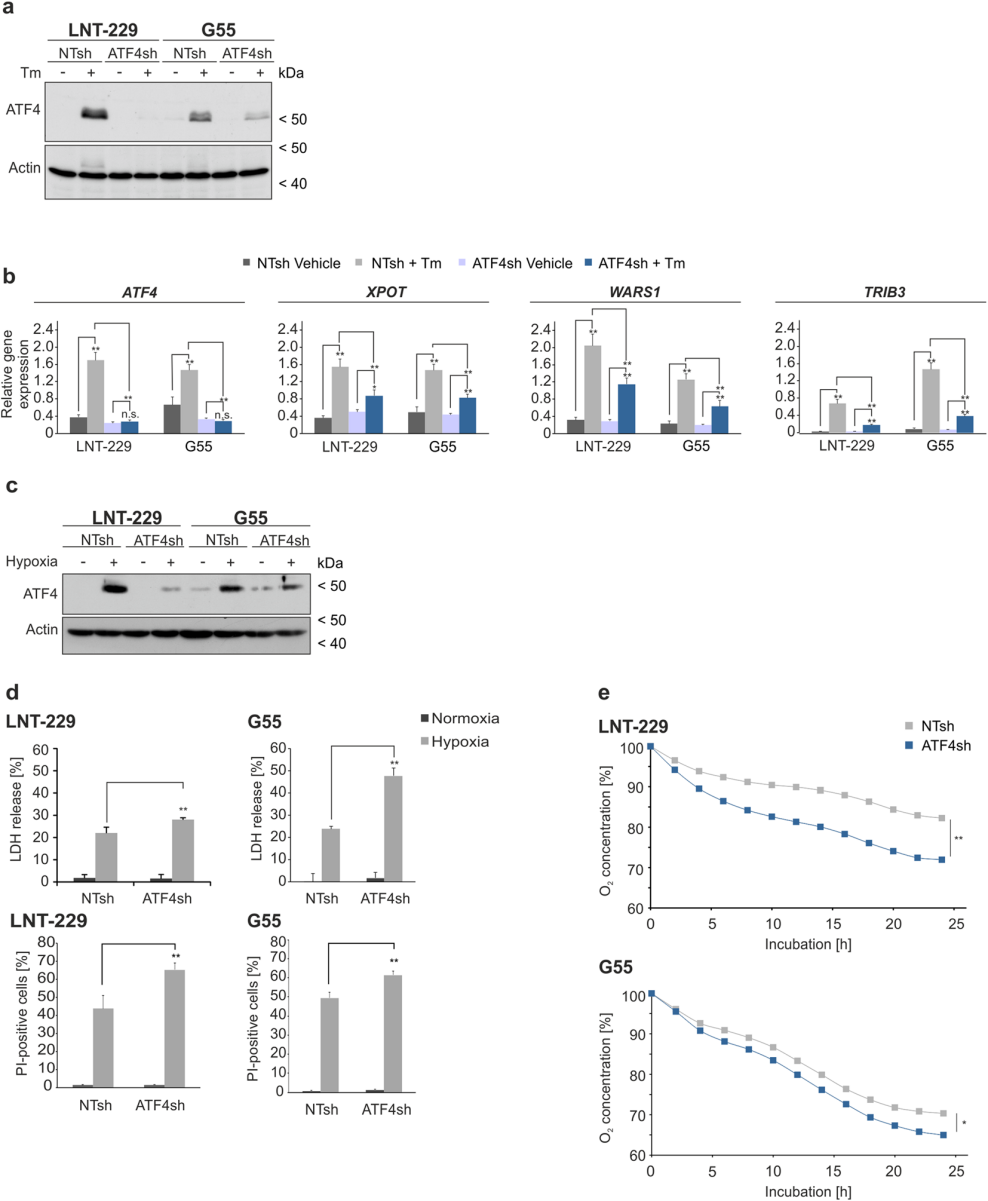
ATF4sh cells are insensitive for ATF4 induction via pharmacological ER stress activation and sensitized to hypoxia-induced cell death. (**a**) LNT-229 and G55 NTsh and ATF4sh cells were incubated with 2 µg/ml tunicamycin (Tm) in serum-free DMEM for 8 h. Cellular lysates were analyzed by immunoblot with antibodies for ATF4 and actin. (**b**) cDNA was analyzed by qPCR with primers for *ATF4*, *XPOT*, *WARS1* and *TRIB3* (n = 3, mean ± SD, n.s. not significant, **p < 0.01, Student’s t-test). *SDHA* and *18S* were used as housekeeping genes for normalization. (**c**) LNT-229 and G55 NTsh or ATF4sh cells were incubated in serum-free DMEM containing 2 mM glucose and 4 mM glutamine in normoxia or hypoxia (0.1% O_2_) for 8 h. Cellular lysates were analyzed for the expression of ATF4 or actin by immunoblot. (**d**) Cells were incubated in serum-free DMEM supplemented with 2 mM glucose and 4 mM glutamine in normoxia or hypoxia (0.1% O_2_). Cell death was analyzed by propidium iodide (PI) uptake and quantified by flow cytometry using BD FACS Diva software (version 6.1.3) (n = 3, mean ± SD, **p < 0.01, Student’s t-test) or by LDH release assay (n = 4, mean ± SD, **p < 0.01, Student’s t-test). (**e**) Oxygen consumption of LNT-229 and G55 NTsh and ATF4sh cells was measured in serum-free DMEM supplemented with 2 mM glucose and 4 mM glutamine overlaid with paraffin oil with a fluorescence-based assay. Oxygen consumption is shown relative to the start of the experiment (n = 3, mean, *p < 0.05, **p < 0.01, Student’s t-test).

To investigate the effects of ATF4 in human GB cell lines LNT-229 and G55 *ATF4* gene suppressed cells (ATF4sh) were generated for further analyses. Stable gene suppression was confirmed via qPCR analysis and revealed a reduction of *ATF4* mRNA in both LNT-229 and G55 ATF4sh cells compared to control cells (Fig. S2a). Tunicamycin dramatically induced ATF4 expression in LNT-229 and G55 control cells (NTsh) whereas no or only a slight effect after pharmacological ER stress induction was observed in ATF4sh cells on protein (Fig. 2a) and mRNA level (Fig. 2b). Glutamine withdrawal resulted in an upregulation of ATF4 protein in LNT-229 control cells but not in the corresponding ATF4 gene-suppressed cells (Fig. S2b). This effect was also observed to a lesser extent in G55 cells most likely due to a lesser gene suppression efficiency of G55 ATF4sh cells (Fig. S2b). Glucose restriction alone did not result in an ATF4 induction under all tested conditions in both control and *ATF4* gene-suppressed cells.

### *ATF4* gene suppression sensitizes human GB cells to hypoxia-induced cell death and increases oxygen consumption

The GB tumor microenvironment is characterized by limited oxygen and glucose supply that drive activation of the cellular stress response machinery which enables cellular adaptation to sustain survival^29^. To analyze the effects of the ER stress response in the context of hypoxia-induced cell death in GB cells we used experimental conditions mimicking the in vivo situation of reduced glucose availability and severe hypoxia^30^. The relevance of ATF4 for adaptation under such conditions was investigated by pharmacological as well as genetic ATF4 modulation. Treatment with tunicamycin which induces ATF4 as shown in Fig. 1a, conferred protection towards combined hypoxia- and nutrient depletion-induced cell death in LNT-229 and G55 cells (Fig. S2c). Immunoblot analysis showed a strong induction of ATF4 only in LNT-229 control (NTsh) and only a reduced induction of ATF4 in LNT-229 ATF4sh cells after treatment under hypoxic, glucose restricted conditions (Fig. 2c). Both, LNT-229 and G55 ATF4sh cells were sensitized to hypoxia-/nutrient depletion-induced cell death compared to control cells (Fig. 2d). In line with these findings, LNT-229 and G55 ATF4sh cells displayed an increased oxygen consumption compared to NTsh cells (Fig. 2e). Conversely, treatment with the ISR inducer tunicamycin led to a reduced oxygen consumption in LNT-229 and G55 cells (Fig. S2d).

### ATF4 gene suppression sensitizes GB cells to temozolomide

**Figure 3:**
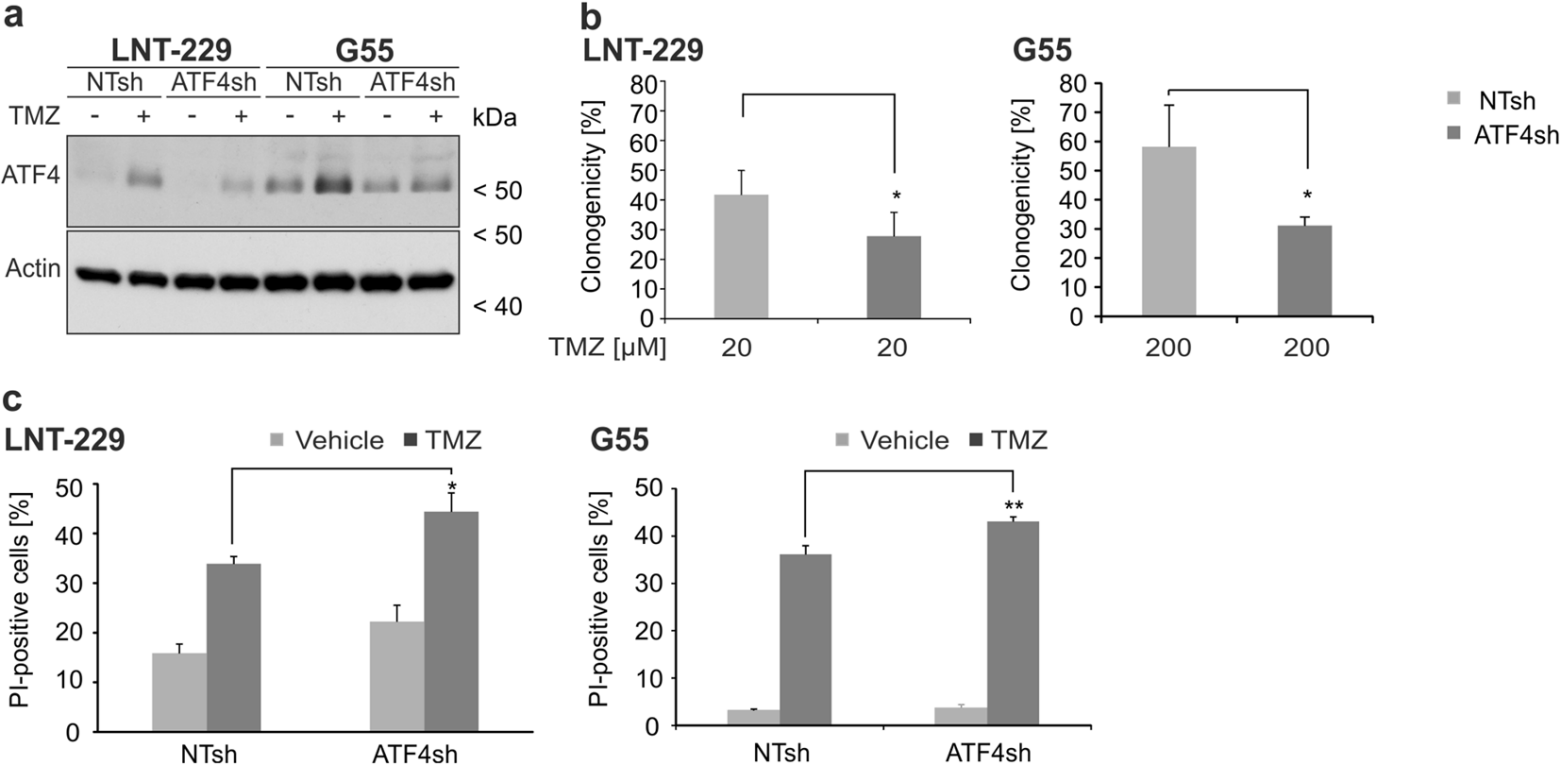
Gene suppression of *ATF4* sensitizes human GB cells to temozolomide induced stress. (**a**) LNT-229 and G55 control (NTsh) or *ATF4* knockdown (ATF4sh) cells were treated with 400 µM temozolomide (TMZ) in serum-free DMEM for 8 h. Cellular lysates were analyzed by immunoblot with antibodies for ATF4 and actin. (**b**) LNT-229 and G55 NTsh and ATF4sh cells were treated with 20 µM TMZ or 200 µM TMZ respectively for 24 h and were further allowed to grow in DMEM for 5 days. Clonogenicity is shown relative to vehicle (DMSO) (n = 3, mean ± SD, *p < 0.05, Student’s t-test). (**c**) LNT-229 and G55 NTsh and ATF4sh cells were treated with 800 µM TMZ for 72 h in serum-free DMEM. Cell death was analyzed by propidium iodide (PI) staining and quantified by flow cytometry (n = 3, mean ± SD, *p < 0.05, **p < 0.01, Student’s t-test). Data were analyzed with BD FACS Diva software (version 6.1.3).

To investigate a potential effect of temozolomide on ATF4 expression, experiments with LNT-229 and G55 ATF4sh and corresponding NTsh cells were performed. In both cell lines, ATF4 expression was increased to a smaller extent in ATF4sh cells on protein level (Fig. 3a). In line with these results, clonogenicity assays revealed that LNT-229 and G55 ATF4sh cells displayed a reduced clonal survival confirming a higher sensitivity towards temozolomide compared to the corresponding NTsh control (Fig. 3b). Furthermore LNT-229 ATF4sh cells showed an enhanced susceptibility to temozolomide treatment (Fig. 3c).

### Pharmacological inhibition of ATF4 activation sensitizes GB cells to hypoxia induced cell death and increases temozolomide susceptibility

To further investigate the effect of ISR inhibition in the context of hypoxia induced cell death and to evaluate whether ISR inhibition could improve temozolomide treatment, we employed the pharmacological ISR inhibitor ISRIB.

**Figure 4:**
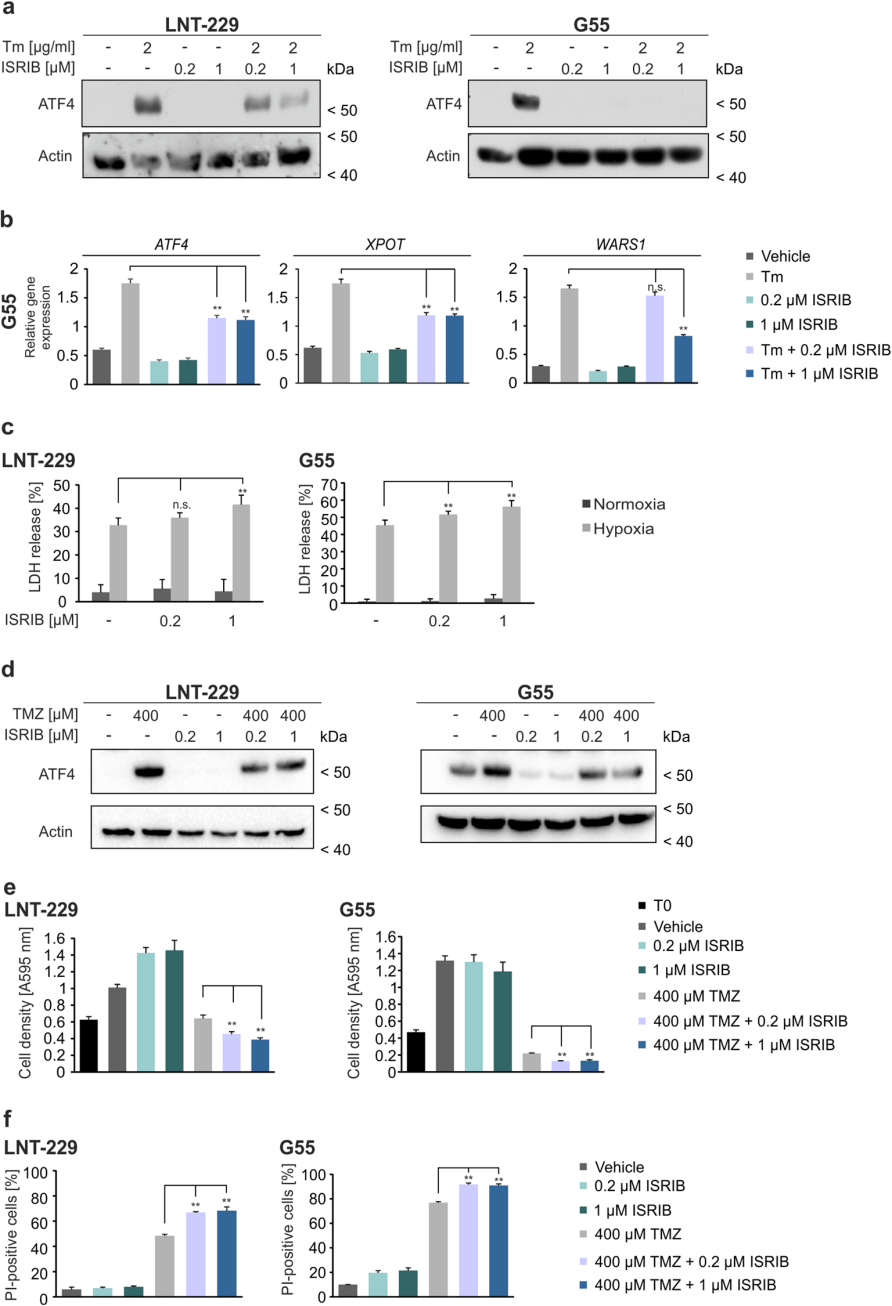
ISRIB treatment sensitizes human GB cells to hypoxia induced cell death and increases temozolomide toxicity. (**a**) LNT-229 and G55 cells were treated with 2 µg/ml tunicamycin (Tm), 0.2 µM or 1 µM ISRIB or a combination of both substances in serum-free DMEM for 8 h as indicated. Cellular lysates were analyzed by immunoblot with antibodies for ATF4 and actin. (**b**) Isolated cDNA of G55 cells was analyzed by qPCR for the expression of *ATF4*, *XPOT* and *WARS1*. *SDHA* and *18S* were used for normalization (n = 3, mean ± SD, n.s. not significant, **p < 0.01, Student’s t-test). (**c**) LNT-229 and G55 cells were incubated in serum-free DMEM without glutamine in normoxia or hypoxia (0.1% O_2_). Cell death was analyzed by LDH release assay (n = 4, mean ± SD, n.s. not significant, **p < 0.01, Student’s t-test). (**d**) Cellular lysates of LNT-229 and G55 cells treated with temozolomide and ISRIB for 24 h in serum-free DMEM as indicated were analyzed by immunoblot with antibodies for ATF4 and actin. (**e**) LNT-229 and G55 cells were treated for 72 h in serum-free DMEM as indicated. Cell densities were measured by CV staining (n = 3, mean ± SD, **p < 0.01, Student’s t-test). (**f**) Cell death of LNT-229 and G55 cells was analyzed by propidium iodide (PI) uptake and quantified by flow cytometry (n = 3, mean ± SD, **p < 0.01, Student’s t-test) after treatment with temozolomide and ISRIB in serum-free DMEM for 72 h as indicated. Data analysis was performed with BD FACS Diva software (version 6.1.3).

While ISRIB treatment did not influence ATF4 expression in LNT-229 and G55 cells, co-treatment with tunicamycin and ISRIB resulted in decreased ATF4 expression on protein level compared to cells treated with tunicamycin alone (Fig. 4a). Similar results were observed on mRNA level of target genes where combined treatment with both substances resulted in reduced translation of *ATF4* itself and *ATF4* target genes *XPOT* and *WARS1* (Fig. 4b). Under glucose restricted, hypoxic conditions, where *ATF4* gene suppression resulted in increased cell death (Fig. 2d), ISR inhibition with ISRIB similarly sensitized LNT-229 and G55 cells to hypoxia induced cell death (Fig. 4c).

Temozolomide induced ATF4 activation was reduced in LNT-229 and G55 cells when ISRIB was present (Fig. 4d). ISRIB treatment did not influence cell growth in LNT-229 and G55 cells after 72 h, but in combination with temozolomide it further decreased cell density compared to treatment with temozolomide alone (Fig. 4e). Furthermore, ISR inhibition with ISRIB significantly increased temozolomide susceptibility in LNT-229 and G55 cells after 72 h (Fig. 4f) in line with our results that ATF4 is important for resistance against temozolomide (Fig. 3b,c).

## Discussion

Adaptation to the conditions of the tumor microenvironment is a central challenge for GB cells in order to enable survival and proliferation. ER stress and amino acid deprivation, frequently present in the GB microenvironment, trigger activation of the transcription factor ATF4 which induces a cellular adaptive stress response. Therefore, ATF4 is a logical candidate for therapeutic inhibition to enhance the susceptibility of GB cells towards existing therapy approaches.

Employing our paradigm of hypoxia-induced cell death with severe hypoxia and glucose restriction^30^, we here provide evidence that supports an important role of ATF4 and the ISR to cope with conditions found in the GB microenvironment (Fig. S3). ATF4 was induced under hypoxic conditions and pharmacological ER stress induction in human GB cells (Figs. 1a, 2c). Gene suppression of *ATF4* sensitized GB cells to hypoxia induced cell death (Figs. 2c,d, S2c) indicating a protective role of ATF4 expression and induction of the ISR. In line, we found an increased oxygen consumption in ATF4sh cells indicating that these cells are more dependent on oxygen (Fig. 2e). This concept that a higher oxygen consumption correlates with enhanced sensitivity to hypoxia-induced cell death has previously been reported for *PPARGC1A* (PGC-1α) overexpressing GB cells^31^. In contrast, pharmacological ATF4 induction with tunicamycin resulted in a reduced oxygen consumption compared to vehicle treated LNT-229 and G55 cells (Fig. S2d) indicating that GB cells with an intact ISR reduce their oxygen consumption during ER stress conditions. Interestingly, it has recently been reported that the stress responses mediated by the ISR and via mTOR display a significant overlap in their translational programs^32^. In line with this finding, we have previously shown that a defective mTOR inhibition sensitizes GB cells to hypoxia-induced cell death^33^ in a similar manner as a defective ISR (Fig. 2d). In parallel, dysfunction of both stress responses was associated with an increased oxygen consumption (Fig. 2e)^33^.

Pharmacological ER stress induction with tunicamycin and thapsigargin is known to induce ATF4 and a number of ATF4 target genes^8,25–28^. In our experiments, treatment with tunicamycin as well as thapsigargin led to a significant increase of ATF4 and its target genes in human GB cell lines (Fig. 1a,b). This effect was strongly reduced in ATF4sh cells compared to control cells in LNT-229 and G55 (Fig. 2a,b). Furthermore, we could demonstrate that ATF4 was induced by glutamine depletion in GB cells whereas glucose starvation alone did not modulate ATF4 expression (Fig. S1c,d). This finding is consistent with data showing an upregulation of ATF4 via the GCN2/P-eIF2α/ATF4 axis during glutamine starvation conditions in colorectal adenocarcinoma and fibrosarcoma cell lines^10^. Glutamine is an important non-essential amino acid used by cancer cells not only for the synthesis of nucleotides and proteins but also as an energy source for ATP production and to support glutathione biosynthesis in redox stress conditions^34^. It has been reported that glutamine depletion led to a 50% reduction in survival in ATF4sh cells compared to a 25% reduction in control cells^10^. Our results emphasize the role of glutamine depletion in the context of the ISR in human GB cells where an ATF4 upregulation possibly helps the tumor cells to adjust to altered nutrient availability.

Previous studies in human GB cells have shown that ATF4 expression is induced by the alkylating chemotherapeutic agent temozolomide^18,20^. In our experiments we found an ATF4 dependent ISR activation after temozolomide treatment in human GB cell lines (Fig. 1c,d) which was strongly reduced in ATF4sh cells (Fig. 3a). Furthermore, gene suppression of *ATF4* led to a reduction of clonogenicity after temozolomide treatment in LNT-229 and G55 cells compared to the corresponding control cells (Fig. 3b). The fact that the impact of temozolomide treatment on the clonogenicity is more pronounced in G55 cells could be due to the higher basal level of *ATF4* (Fig. S1a) and therefore a higher dependency of those cells on ATF4 activity. ATF4sh cells were also more susceptible to temozolomide induced acute cell death compared to control cells (Fig. 3c). Thus, our results demonstrate that initiation of the ISR and induction of ATF4 are essential for the activation of cellular resistance mechanisms which allow GB cells to overcome temozolomide toxicity (Fig. S3). In previous studies it has been demonstrated that PERK inhibition can be beneficial for cancer treatment^14^. While many ISR inhibitors target PERK itself, ISRIB specifically inhibits the stress response downstream of PERK by reversing the attenuation of eIF2B by phosphorylated eIF2α^15–17^. PERK inhibition was reported to lead to side effects in in vivo models, especially on pancreatic function, whereas targeting phosphorylated eIF2α with ISRIB does not show pancreatic toxicity^14,35^. ISRIB treatment sensitized GB cells to hypoxia induced cell death (Fig. 4c), indicating that ISR inhibition leads to an increased vulnerability to conditions of the tumor microenvironment (hypoxia, nutrient deprivation) and impaired tumor cell survival. Furthermore, our experiments using combined temozolomide and ISRIB treatment highlight the importance of the ISR and especially ATF4 induction for temozolomide resistance in GB cells. ISR inhibition enhanced temozolomide susceptibility in GB cell lines (Fig. 4e,f).

While the ISR inducing agents thapsigargin and tunicamycin are frequently used to study ATF4-mediated cellular effects, these substances are certainly not specific for ATF4. Also with regard to shRNA approaches off-target effects cannot be ruled out. Nevertheless, our work provides consistent evidence that supports an important role of eIF2α-ATF4 axis and the integrated stress response of GB cells to conditions found in the tumor microenvironment (low glucose and hypoxia) and in response to temozolomide.

Analyses of databases suggested elevated ATF4 expression in gliomas in comparison to normal brain tissue and an association of increased *ATF4* transcription with poor overall survival in glioma cohorts^18,36^. Several in vitro investigations have implicated ATF4 in tumor cell survival with sometimes opposing cytoprotective and cytotoxic roles depending on context: ATF4 has been shown to attenuate ferroptosis under dihydroartemisinin-induced ER stress or sorafenib treatment to sustain glioma cell survival and inhibition of this axis was therefore proposed as a therapeutic strategy^37^. On the contrary, another study in GB cells demonstrated that ATF4 played a key role to induce autophagic cell death via reticulophagy (i.e. the selective degradation of the endoplasmic reticulum) in response to loperamide treatment and in this context activation of ATF4 was suggested to be beneficial for tumor therapy^38^. These studies highlight the ambiguous character of the ATF4 mediated stress response depending on the experimental context. A more in depth understanding of ATF4 effects is therefore urgently needed before advancing ATF4 targeting strategies to clinical trials. In our study we approximated in vivo conditions both with regard to metabolic (oxygen and glucose deprivation) and treatment (with temozolomide as the standard chemotherapeutic for gliomas) scenarios. In both scenarios ATF4 mediated protective adaptation in GB cells. A next step is to validate the advantages of a combined temozolomide and anti-ATF4 (e.g. ISRIB treatment) approach in in vivo GB mouse models.

Our preclinical results have potentially high translational value. Primary or secondary resistance to temozolomide is a major problem in GB treatment. Several scenarios of combining temozolomide with an ISR inhibitor (e.g. ISRIB) could be of interest: including ISR inhibition directly in the first line temozolomide treatment algorithm could enhance temozolomide efficacy particularly in MGMT-promoter methylated tumors and maybe also help achieve some efficacy in MGMT unmethylated tumors. Another scenario could be to include ISR inhibition as a treatment concept to (re-)sensitize GB to temozolomide in the recurrent disease setting. In this context, it would be interesting to analyze histology specimens of recurrent gliomas after temozolomide treatment for activation of the ISR which would be a rationale for such an approach in the recurrent disease setting.

The ISR has gained interest across the cancer field and it is likely that candidates for clinical testing will become available. Therapeutic inhibition could be a promising clinical strategy to improve treatment of GBs.

## Material and methods

### Reagents, cell lines and culture conditions

Tunicamycin and thapsigargin were purchased from Tocris (Bristol, UK). All reagents not specified elsewhere were purchased from Sigma (Taufkirchen, Germany). LNT-229 cells were a kind gift of N. de Tribolet (Lausanne, Switzerland). LNT-229 cells were authenticated using STR analysis by Multiplexion (Heidelberg, Germany). The STR profile of the tested cells matched with the known profile for LN-229. LNT-229 and LN-229 cells only differ in their p53 status^39^. G55 T2 cells (named G55 for simplicity throughout the manuscript) were a kind gift of Manfred Westphal and Kathrin Lamszus (Hamburg, Germany)^40^, a STR profile for this cell line has not been deposited in databases yet. MGMT, IDH, p53 and PTEN status of LNT-229 and G55 cells have been described previously and are listed (Table 1)^33,41^.

**Table 1:** Genetic background of LNT-229 and G55 cells.

	MGMT	IDH	p53	PTEN
LNT-229	Methylated	Wildtype	Wildtype	Wildtype
G55	Unmethylated	Wildtype	Mutant	n.a

MGMT, IDH, p53 and PTEN status of G55 and LNT-229 cells^33^.

All cell lines were regularly checked for mycoplasma contamination throughout the study and only contamination-free cells were used for experiments.

Cell lines were cultured in Dulbecco´s modified Eagle´s medium (DMEM) containing 10% fetal calf serum (Biochrom KG, Berlin, Germany), 100 IU/ml penicillin and 100 µg/ml streptomycin (Life Technologies, Karlsruhe, Germany) in cell culture incubators at 37 °C and 5% CO_2_^42^. For experiments comparing sub-cell lines, equal cell densities at the start of the experiment were confirmed by crystal violet (CV) staining in parallel assays^42^.

Human primary GB cells NCH690, NCH421K and NCH644 were purchased from CLS (Eppelheim, Germany) and were cultured in Neurobasal A medium (Thermo Fisher Scientific, Dreieich, Germany) supplemented with 1 × B27 supplement (Thermo Fisher Scientific, Dreieich, Germany), 2 mM glutamine (Thermo Fisher Scientific, Dreieich, Germany), 1U/ml heparin, 100 IU/ml penicillin and 100 µg/ml streptomycin (Life Technologies, Karlsruhe, Germany) and 20 ng/ml EGF and FGF-2 (ReliaTech GmbH, Wolfenbüttel, Germany). Human astrocytes were purchased from Innoprot (Derio, Spain) and were cultured in Astrocyte medium (Innoprot, Derio, Spain) according to the manufacturer’s protocol.

### Temozolomide treatment

The different temozolomide concentrations were chosen based on the experimental setup and cell line characteristics (i.e. MGMT promoter methylation status). The effect of low doses of temozolomide on clonal survival are a known phenomenon and are usually by several factors lower than doses required to induce acute toxicity^43,44^. For clonal survival analyses of MGMT promoter methylated LNT-229 cells 20 µM temozolomide and for MGMT promoter unmethylated G55 cells 200 µM temozolomide were chosen. For acute toxicity cells were treated with higher doses of temozolomide for 72 h. To investigate acute effects of temozolomide treatment on mRNA expression and protein levels, cells were incubated with 400 µM temozolomide for 8 h.

### Generation of *ATF4* gene suppressed cells

pLKO.1 plasmids targeting *ATF4* (ATF4sh, TRCN0000329695) and non-targeting control (NTsh, #1864) were purchased from Sigma and Addgene respectively. HEK293 cells were used for lentiviral production with pCMV-dR8.2 dvpr (Addgene, #8455) and pCMV-VSVG (Addgene, #8454) as packaging and envelope plasmids according to the manufacturer’s protocol. Lentiviral transduction was performed using polybrene (Merck Millipore, Darmstadt, Germany). ATF4sh and NTsh cells were cultured in standard medium supplemented with 2 µg/ml puromycin (AppliChem, Darmstadt, Germany). Experiments were performed with early passage pooled clones.

### Induction of hypoxia

Cells were seeded and allow to attach overnight under normoxia. Experiments were performed in serum-free DMEM containing 2 mM glucose and 4 mM glutamine under normoxia or 0.1% O_2_ hypoxia in BD GasPak pouches (Becton–Dickinson, Heidelberg, Germany) for anaerobic culture for the indicated intervals^33,45^.

### Immunoblot

Cell lysate preparation and immunoblot analysis was performed as described previously^33^. Primary antibodies for ATF4 (Proteintech, Rosemont, IL, USA, 10835-1-AP and Cell Signaling Technology, Danvers, MA, USA, #11815) or actin (Santa Cruz Biotechnology, Santa Cruz, CA, USA, #sc-1616) were employed as indicated. Secondary anti-rabbit and anti-goat antibodies were purchased from Jackson ImmunoResearch (West Grove, PA, USA, #111-036-144) and Santa Cruz (#sc-2020). Chemiluminescence was used for detection. All full length immunoblots are shown in Fig. S4.

### RNA extraction and quantitative PCR analysis

Total RNA was extracted using TRIzol and EXTRACTME RNA isolation kit (Blirt, Gdansk, Poland). cDNA was synthesized using the Vilo cDNA synthesis kit (Invitogen, Carlsbad, CA, USA)^33^. Quantitative RT-PCR (qPCR) was performed using the Absolute Blue SYBR Green Fluorescein q-PCR Mastermix (Thermo Fisher Scientific, Dreieich, Germany) with the corresponding primer pairs (Table S1). *18S* and *SDHA* were used as housekeeping genes for normalization. Data was analyzed using the Vandesompele method^46^.

### Cell density and viability assays

For cell growth analyses cells were seeded in 96-well plates and allowed to attach overnight. After treatment, cell density was analyzed by CV staining after the indicated intervals as previously described^42,47^. Cell viability was analyzed by lactate dehydrogenase release assays with the Cytotoxicity Detection Kit (LDH) (Roche, Mannheim, Germany) or by propidium iodide (PI) uptake assays via flow cytometry as previously described^30,33^. Data were acquired with a BD Canto II flow cytometer and analyzed with BD FACS Diva software (version 6.1.3).

### Clonal survival assays

For analysis of clonal survival 500 cells were seeded per well of a 6 well plate and allowed to attach overnight. Cells were treated with temozolomide in serum-free DMEM. Medium was replaced with fresh DMEM containing 10% FCS after 24 h. CV staining was performed 5 days later. Clones were counted manually^48^.

### Oxygen consumption

Cells were seeded in 24-well plates with oxygen sensors (PreSens, Regensburg, Germany) and allowed to attach overnight. Medium was replaced and cells were overlaid with sterile paraffin oil. Oxygen consumption was measured in optical sensor plates and concentrations of remaining oxygen were recorded over time^33^. For statistical analysis values of the end-points of the experiments were analyzed by a two-tailed student’s t-tests.

### Statistics

Quantitative data are expressed as mean and standard deviation (SD). A two-tailed student’s t-test was used for the calculation of p-values. Values of p < 0.01 were considered as highly significant, p < 0.05 as significant and p > 0.05 as not significant (n.s.).

## Supplementary Information

Supplementary Information.

## Data Availability

The datasets used or analyzed during the current study are available from the corresponding author on reasonable request.
