# Supplementary material for: Activating transcription factor 4 mediates adaptation of human glioblastoma cells to hypoxia and temozolomide
Source: Sci Rep. 2021 Jul 8;11:14161. doi: 10.1038/s41598-021-93663-1 (PMC8266821; doi:10.1038/s41598-021-93663-1)
Supplement: Supplementary file 1 — Supplementary Information. [file 41598_2021_93663_MOESM1_ESM.docx]

**Supplementary Information**

**Activating transcription factor 4 mediates adaptation of human glioblastoma cells to hypoxia and temozolomide**

Nadja I. Lorenz^1-4 *^, Alina C.M. Sittig^1-4 *^, Hans Urban^1-4^, Anna-Luisa Luger^1-4^, Anna L. Engel^1-4^, Christian Münch^3,5,6^, Joachim P. Steinbach^1-4^ and Michael W. Ronellenfitsch^1-4^

^1^ Dr. Senckenberg Institute of Neurooncology, University Hospital Frankfurt, Goethe University, Frankfurt am Main, Germany

^2^ German Cancer Consortium (DKTK), Partner Site Frankfurt/Mainz, Frankfurt am Main, Germany

^3^ Frankfurt Cancer Institute (FCI), University Hospital Frankfurt, Goethe University, Frankfurt am Main, Germany

^4^ University Cancer Center Frankfurt (UCT), University Hospital Frankfurt, Goethe

University, Frankfurt am Main, Germany

^5^ Institute of Biochemistry II, Goethe University, Frankfurt am Main, Germany

^6^ Cardio-Pulmonary Institute, Frankfurt am Main, Germany

* These authors contributed equally to this work.

Correspondence to: M.Ronellenfitsch@gmx.net

**List of supporting data:**

Supplementary Table 1: Primer pairs for qPCR analysis

Supplementary Figure 1: Pharmacological ER stress activation results affects proliferation in human GB cells

Supplementary Figure 2: ATF4 regulates sensitivity to hypoxia in human GB cell lines

Supplementary Figure 3: Effects of ATF4 activation in NTsh and ATF4sh cells

Supplementary Figure 4: Fig. S4 Full-length images of immunoblots.

**Supplementary Experimental Procedure**

**Table S1: Primer pairs for qPCR analysis**

| **Gene** | **Primer pair** |
| --- | --- |
| ***ATF4*** | fw: 5′-ATGACCGAAATGAGCTTCCTG-3′  rev: 5’- GCTGGAGAACCCATGAGGT-3’ |
| ***WARS1*** | fw: 5’- AGCACCTACCAGTAATCATGGC-3’  rev: 5’- TCCAAACCGAACAATGAGCTT-3’ |
| ***XPOT1*** | fw: 5’- AGGGAGACGCTCATATCATGG-3’  rev: 5’- TTGGGCGGCTTTATTTCGTAT-3’ |
| ***TRIB3*** | fw: 5’- AAGCGGTTGGAGTTGGATGAC-3’  rev: 5’- CACGATCTGGAGCAGTAGGTG-3’ |
| ***18S*** | fw: 5’- CGGCTACCACATCCAAGGAA-3’  rev: 5’- GCTGGAATTACCGCGGCT-3’ |
| ***SDHA*** | fw: 5’-TGGGAACAAGAGGGCATCTG-3’  rev: 5’-CCACCACTGCATCAAATTCATG-3’ |

**Supplementary Figures**

**
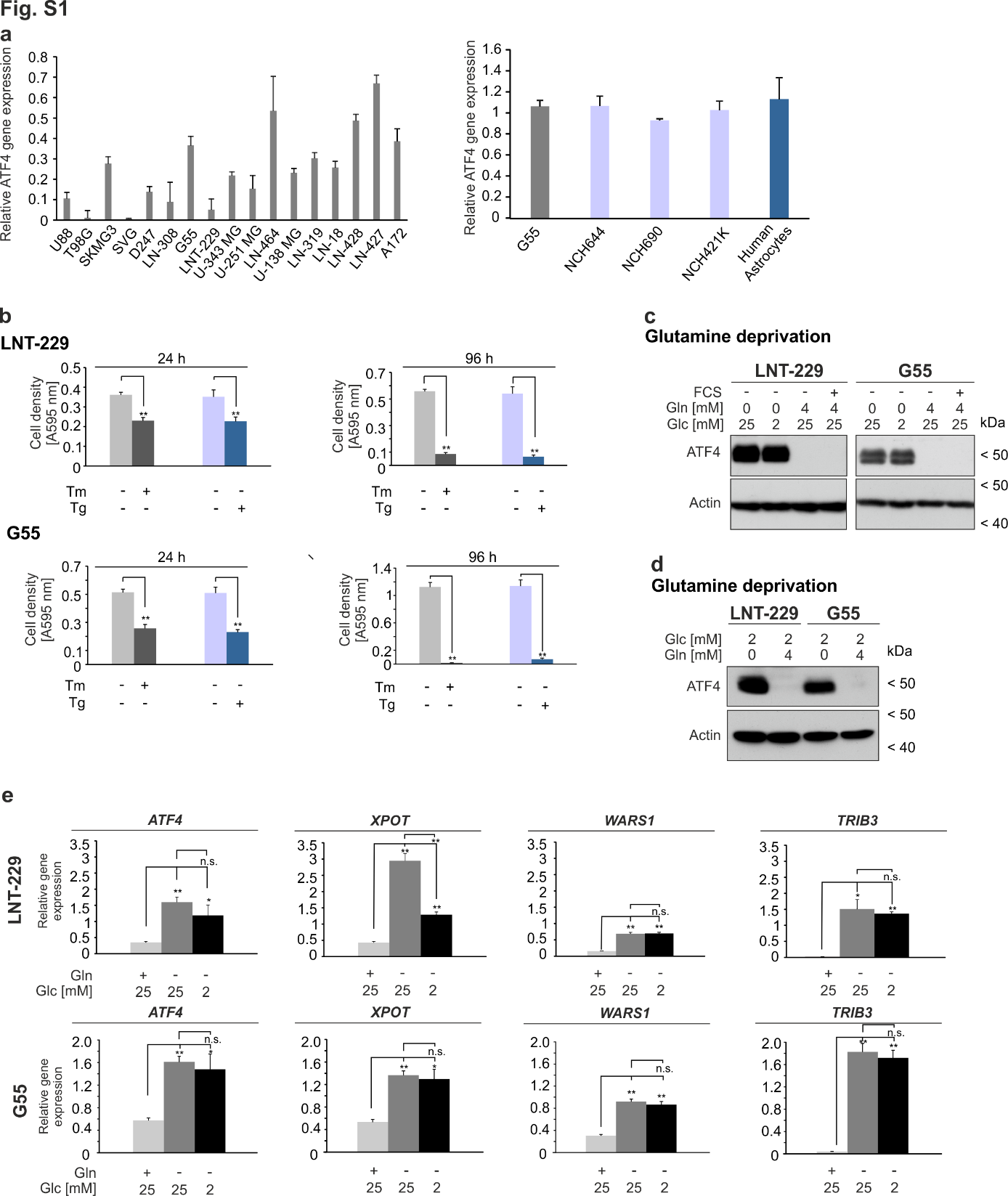
**

**Fig. S1 Pharmacological ER stress activation results affects proliferation in human GB cells.**

(a) Basal ATF4 mRNA levels of a panel of human glioma cell lines, primary GB cells (NCH690, NCH644 and NCH421K) as well as human astrocytes under standard conditions were analyzed by qPCR. *SDHA* and *18S* were used as housekeeping genes for normalization. (b) LNT-229 and G55 cells were treated with 2 µg/ml tunicamycin (Tm) or 1 µM thapsigargin (Tg) for the indicated interval. Cell densities were measured by CV staining (n=3, mean ± SD). (c,d) Cellular lysates of LNT-229 and G55 cells treated with DMEM without or with 10% FCS supplemented with 2 or 25 mM glucose (Glc) and 0 or 4 mM glutamine (Gln) for 8 h were analyzed by immunoblot with antibodies for ATF4 and actin. (e) LNT-229 and G55 cells were treated in serum-free DMEM containing 4 mM glutamine (Gln) and 2 mM or 25 mM glucose (Glc) for 8 h as indicated. QPCR was used to analyze isolated cDNA for the expression of *ATF4*, *XPOT*, *WARS1* and *TRIB3*. Values were normalized to *18S* and *SDHA* gene expression (n=3, mean ± SD).


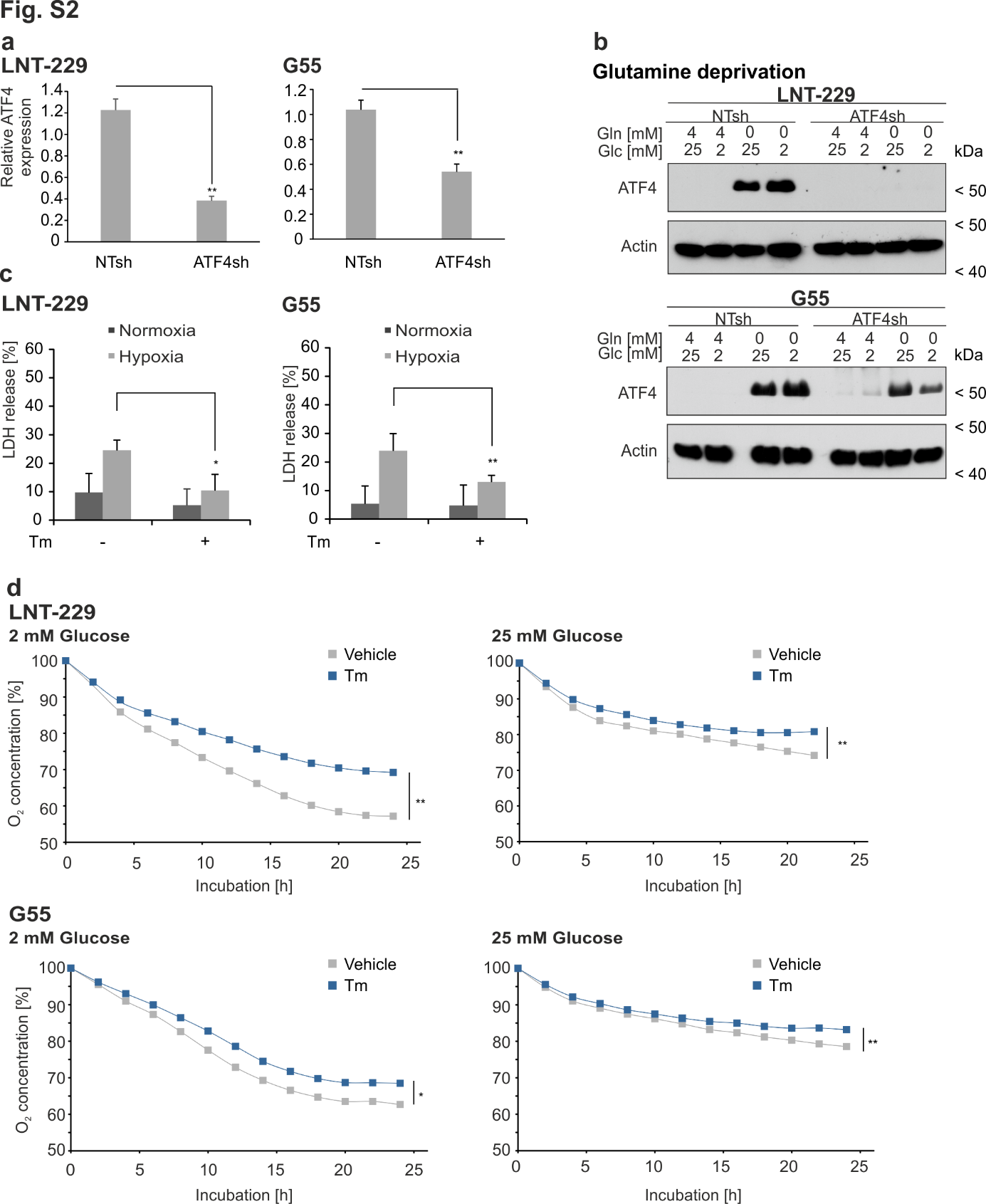


**Fig. S2 ATF4 regulates sensitivity to hypoxia in human GB cell lines.**

(a) LNT-229 and G55 cells were stably transfected with non-targeting control (NTsh) or ATF4 knockdown (ATF4sh) plasmids. cDNA was checked for ATF4 knockdown by qPCR with primers for *ATF4*. Values were normalized to *SDHA* and *18S* housekeeping gene expression (n=3, mean ± SD, ** p < 0.01, Student’s t-test). (b) LNT-229 or G55 NTsh or ATF4sh cells were treated in serum-free DMEM with or without 4 mM glutamine and 2 or 25 mM glucose for 8 h as indicated. Cellular lysates were analyzed for the expression of ATF4 and actin by immunoblot. (c) LNT 229 and G55 cells were pre-incubated in serum-free DMEM containing 25 mM glucose and 2 µg/ml tunicamycin or vehicle (DMSO) for 4 h. Afterwards medium was replaced by serum-free DMEM containing 2 mM glucose and 4 mM glutamine with or without 2 µg/ml tunicamycin. Cells were incubated in normoxia or hypoxia (0.1% O2). Cell death was analyzed by LDH release assay (n=4, mean + SD, * p < 0.05, ** p < 0.01, Student’s t-test). (d) Oxygen consumption of LNT-229 and G55 cells treated with 2µg/ml tunicamycin or vehicle (DMSO) was measured in serum-free DMEM containing 25 mM glucose and 4 mM glutamine or in serum-free DMEM supplemented with 2 mM glucose and 4 mM glutamine overlaid with paraffin oil using a fluorescence-based assay. Oxygen consumption is shown relative to the start of the experiment (n=3, mean + SD, * p < 0.05, ** p < 0.01, Student’s t-test).


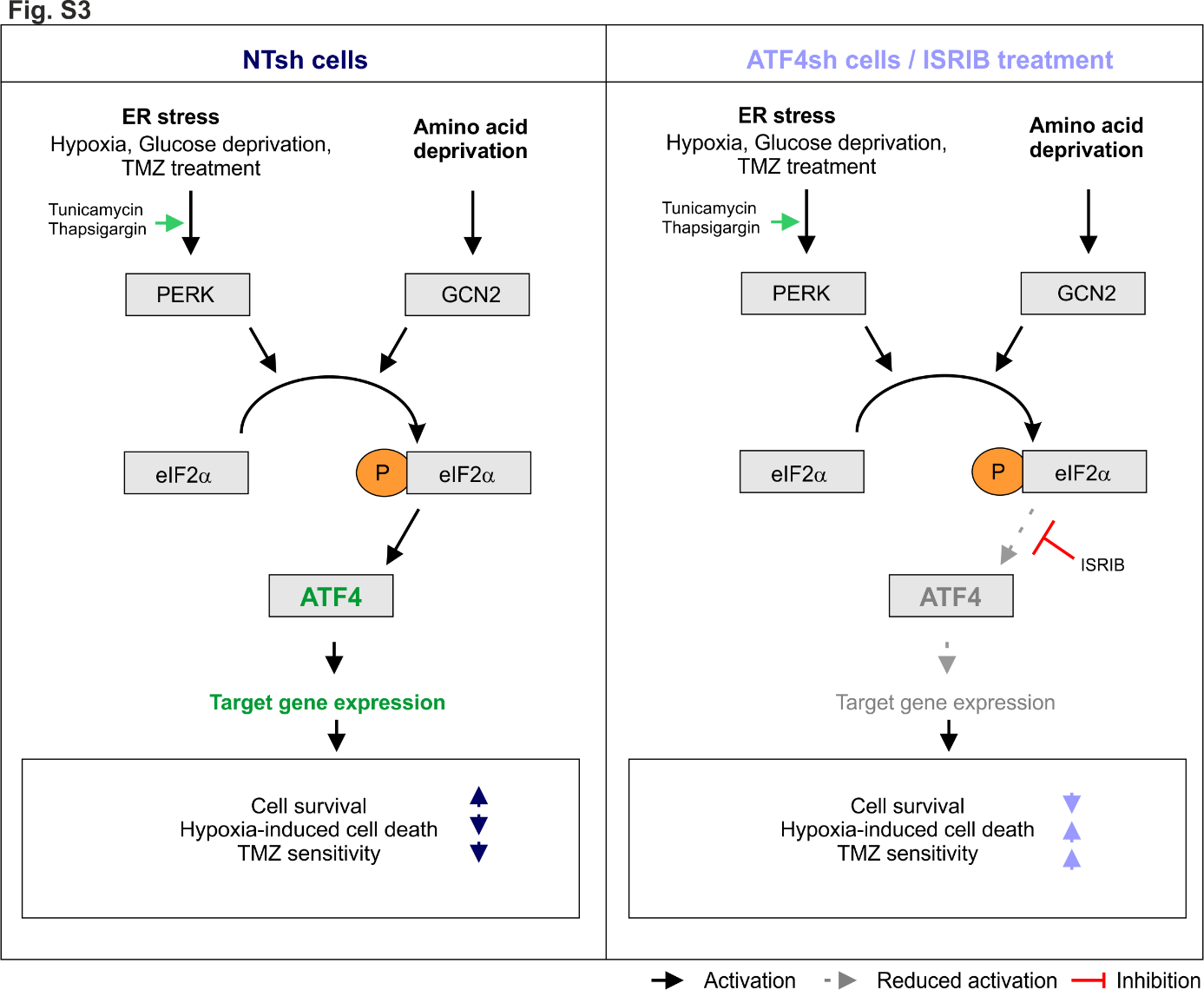


**Fig. S3 Effects of ATF4 activation in NTsh and ATF4sh cells.**

Conditions of the tumor microenvironment (hypoxia, glucose deprivation) as well as temozolomide (TMZ) treatment induce ER stress and activate the ISR via PERK resulting in the phosphorylation of eIF2α. PERK activation can be modulated by the ER stress activators tunicamycin and thapsigargin. Moreover, amino acid deprivation leads to eIF2α phosphorylation in a GCN2 dependent manner. In consequence of eIF2α phosphorylation ATF4 is activated in NTsh (left panel) but only to a strongly diminished extent in ATF4sh cells (right panel). Pharmacological ISR inhibition with ISRIB targets phosphorylated eIF2α (P-eIF2α) and reduces ATF4 induction. ATF4 induces the expression of target genes in NTsh cells to protect from hypoxia-induced cell death, increase cell survival and mediate treatment resistance against TMZ (left panel). In contrast, cells with impaired ATF4 induction e.g. by ATF4 gene suppression (ATF4sh) or ISRIB treatment are sensitized to such conditions (right panel).


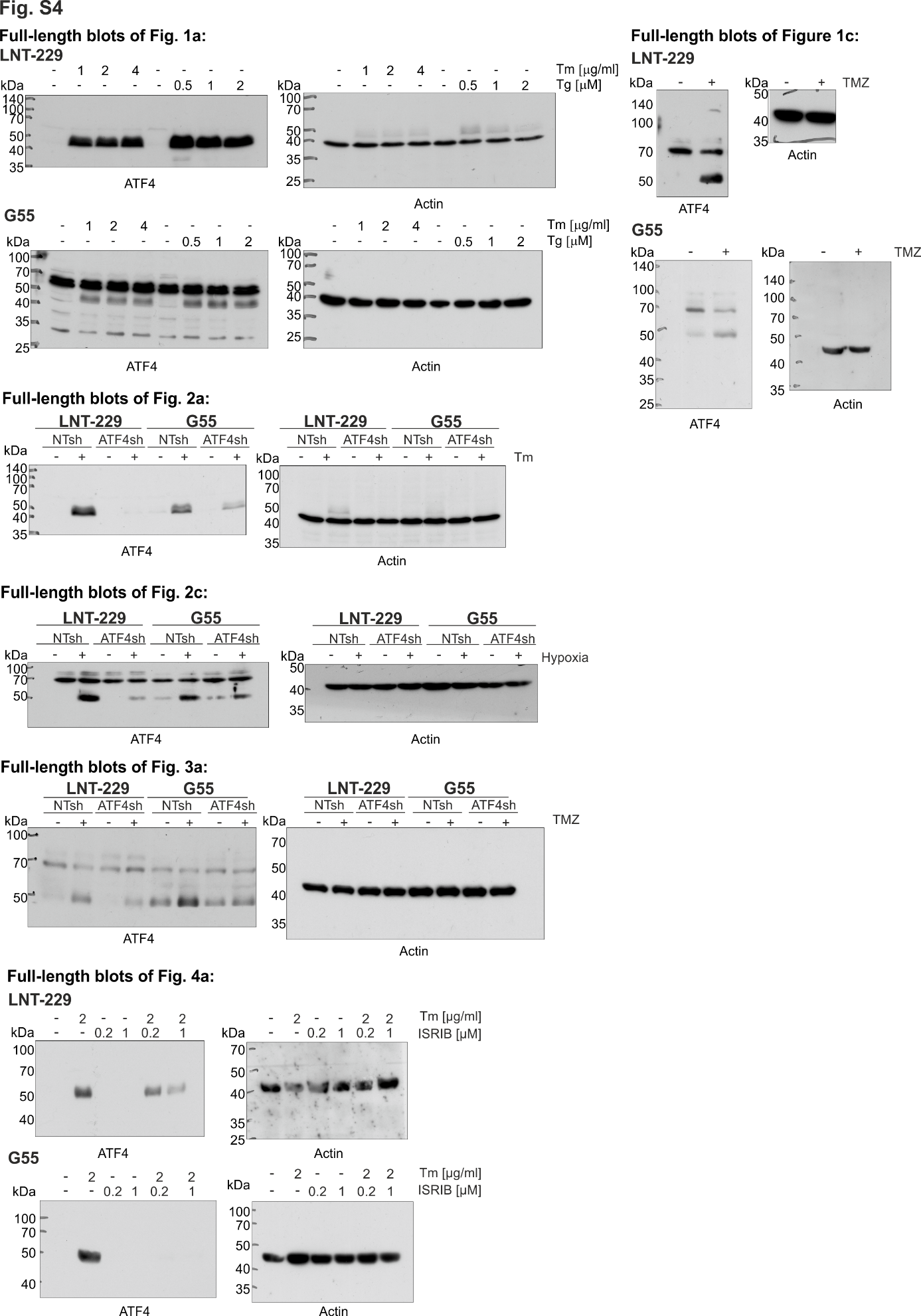


**
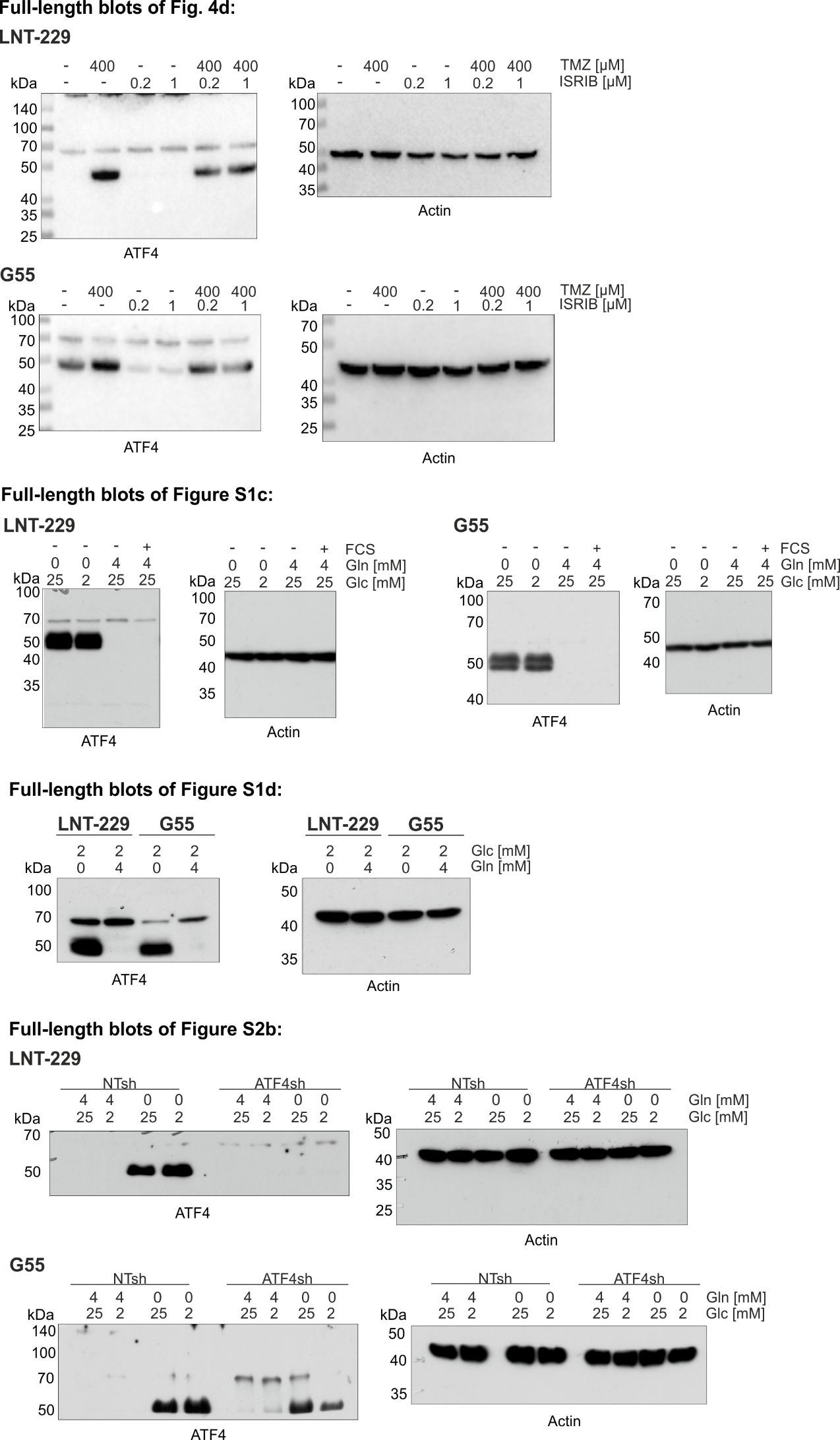
**

**Fig. S4 Full-length images of immunoblots.**
